# Metformin improves salivary gland inflammation and hypofunction in murine Sjögren’s syndrome

**DOI:** 10.1186/s13075-019-1904-0

**Published:** 2019-06-04

**Authors:** Ji-Won Kim, Sung-Min Kim, Jin-Sil Park, Sun-Hee Hwang, JeongWon Choi, Kyung-Ah Jung, Jun-Geol Ryu, Seon-Yeong Lee, Seung-Ki Kwok, Mi-La Cho, Sung-Hwan Park

**Affiliations:** 1 Division of Rheumatology, Department of Internal Medicine, Seoul St. Mary’s Hospital, College of Medicine, The Catholic University of Korea, Seoul, Republic of Korea; 2 Rheumatism Research Center, Catholic Research Institute of Medical Science, College of Medicine, The Catholic University of Korea, Seoul, Republic of Korea; 3 Division of Rheumatology, Department of Internal Medicine, Catholic University of Daegu School of Medicine, Daegu, Republic of Korea

**Keywords:** Metformin, Sjögren’s syndrome, AMP-activated protein kinase, TOR serine-threonine kinase, STAT3, Th17 cells, B-lymphocyte

## Abstract

**Background:**

Activated T and B cells participate in the development and progression of Sjögren’s syndrome (SS). Metformin, a first-line anti-diabetic drug, exerts anti-inflammatory and immunomodulatory effects by activating AMPK. We investigated the therapeutic effect of metformin in non-obese diabetic (NOD)/ShiLtJ mice, an animal model of SS.

**Methods:**

Metformin or vehicle was administered orally to the mice for 9 weeks. The salivary flow rate was measured at 11, 13, 15, 17, and 20 weeks. Histological analysis of the salivary glands from vehicle- and metformin-treated mice was conducted. CD4^+^ T and B cell differentiation in the peripheral blood and/or spleen was determined by flow cytometry. Serum total IgG, IgG1, and IgG2a levels were determined by enzyme-linked immunosorbent assay.

**Results:**

Metformin reduced salivary gland inflammation and restored the salivary flow rate. Moreover, metformin reduced the interleukin (IL)-6, tumor necrosis factor-α, IL-17 mRNA, and protein levels in the salivary glands. Metformin reduced the Th17 and Th1 cell populations and increased the regulatory T cell population in the peripheral blood and spleen and modulated the balance between Tfh and follicular regulatory T cells. In addition, metformin reduced B cell differentiation into germinal center B cells, decreased the serum immunoglobulin G level, and maintained the balance between IL-10- and IL-17-producing B cells.

**Conclusion:**

Metformin suppresses effector T cells, induces regulatory T cells, and regulates B cell differentiation in an animal model of SS. In addition, metformin ameliorates salivary gland inflammation and hypofunction, suggesting that it has potential for the treatment of SS.

**Electronic supplementary material:**

The online version of this article (10.1186/s13075-019-1904-0) contains supplementary material, which is available to authorized users.

## Background

Sjögren’s syndrome (SS) is an immune-related chronic inflammatory disease that typically involves the salivary and lacrimal glands. The pathogenesis of SS involves abnormal innate and adaptive immune responses [1]. Environmental triggers such as viral infections result in the activation of the interferon alpha pathway in the mucosal epithelial cells of individuals with certain genetic factors [2]. The subsequent increase in the levels of B cell-activating factors leads to autoreactive B cell activation and production of autoantibodies. Additionally, the interaction of cognate T and B cells induces a dysregulated B cell response and the formation of ectopic germinal center (GC)-like structures in SS [3]. Few drugs effective against SS are available.

Metformin is an anti-diabetic drug that reduces hepatic gluconeogenesis by AMP-activated protein kinase (AMPK)-dependent and AMPK-independent mechanisms [4–8]. AMPK is a serine/threonine kinase that senses cellular energy levels [6]. AMPK activation by metformin exerts anti-hyperglycemic, anti-inflammatory, and immunomodulatory effects by inhibiting the mechanistic target of rapamycin (mTOR) and signal transducer and activator of transcription (STAT) 3 signaling pathways [9–12].

The mTOR and STAT intracellular signaling pathways modulate the cell cycle and immunity. The mTOR pathway controls cell growth, proliferation, and survival by sensing signals from growth factors, cytokines, and metabolic status [13]. Moreover, mTOR regulates T cell differentiation: mTOR-activated T cells differentiate into effector T cells, whereas mTOR-deficient T cells differentiate into regulatory T (Treg) cells [14]. The STAT pathway mediates cytokine signaling and controls cell growth and apoptosis [15]. In addition, STAT3 participates in T helper 17 (Th17), follicular helper T (Tfh) [16–18], and B cell [19, 20] differentiation.

In SS, T cells are involved in target organ inflammation and promote B cell activation [21, 22]. Th17 and Tfh cells are activated in SS, and the balance between these cell types and their regulatory counterparts [Treg and follicular regulatory T (Tfr) cells] may be important in its pathophysiology [3]. Additionally, the STAT3 pathway is involved in the pathogenesis of SS [23–25]. The peripheral blood mononuclear cells (PBMCs) and salivary glands from patients with SS show abnormally activated STAT3 and increased interleukin (IL)-17 expression, which might be associated with loss of STAT3 suppressor function [23, 26]. Indeed, in a mouse model of SS, an mTOR-targeted drug suppressed autoimmune dacryoadenitis [27]. Therefore, therapeutics targeting these signaling pathways may be effective against SS.

We hypothesized that metformin, which has AMPK-dependent mTOR–STAT3 inhibitory activity, would modulate T and B cell differentiation and reduce salivary gland inflammation in SS. Using non-obese diabetic (NOD)/ShiLtJ mice, an animal model of SS, we examined the effects of metformin on (1) salivary gland inflammation and salivary flow rate, (2) T cell differentiation to effector or regulatory T cells, and (3) B cell differentiation.

## Methods

### Animals

NOD/ShiLtJ mice (aged 7–9 weeks, female) were purchased from The Jackson Laboratory (Bar Harbor, ME, USA). The mice were maintained under specific pathogen-free conditions at the Catholic Research Institute of Medical Science of the Catholic University of Korea and were fed standard mouse chow (Ralston Purina, St. Louis, MO, USA) and water ad libitum. All experimental procedures were examined and approved by the Animal Research Ethics Committee of the Catholic University of Korea (permit number: CUMC-2016-0264-02) and conformed to all applicable US National Institutes of Health guidelines.

All surgeries were performed under isoflurane anesthesia, and all efforts were made to minimize suffering. The experimental protocol was approved, and all animals were treated and sacrificed in accordance with the guidelines of the Catholic University of Korea on the use and care of animals.

### Measurement of salivary flow rate

The mice were anesthetized by isoflurane (2%) inhalation and injected intraperitoneally with pilocarpine (5 mg/kg). Saliva was collected from the oral cavity for 7 min in a microtube. The microtube containing the saliva was briefly centrifuged, and the volume was measured using a micropipette. Salivary flow rate is expressed in microliters per minute per gram of body weight (μL/min/g) [28].

### Metformin treatment

Metformin was obtained from Sigma-Aldrich (St. Louis, MO, USA) and dissolved in saline. Eleven-week-old mice were orally administered 50 mg/kg metformin daily for 9 weeks. A previous study also orally administered 50 mg/kg metformin at a collagen-induced arthritis model to examine the therapeutic effect of metformin [29]. Human equivalent dose is 4 mg/kg for 50 mg/kg mouse dose, after converted by the equation (human equivalent dose = mouse dose × [mouse *K*_*m*_/human *K*_*m*_]; *K*_*m*_, correction factor, estimated by dividing the average body weight to its body surface area) [30]. The control mice were administered vehicle (saline).

### Histological analysis of the salivary gland

Tissues were fixed with 10% neutral buffered formalin for 24 h at 4 °C and then embedded in paraffin. The tissue sections were used for hematoxylin and eosin (H&E) staining. After H&E staining, the sections were examined under a photomicroscope (Olympus, Tokyo, Japan) at × 100 magnification and scored. Histological score was determined as follows [31]: a grade of 1 indicated that 1–5 foci of mononuclear cells were seen (> 20 cells per focus), a grade of 2 indicated that > 5 foci of mononuclear cells were seen without significant parenchymal destruction, a grade of 3 indicated that multiple confluent foci were seen with moderate degeneration of parenchymal tissue, and a grade of 4 indicated extensive infiltration of the gland with mononuclear cells and extensive parenchymal destruction. The score was calculated by an observer (JWC) who was blinded to the experimental group. Three fields per tissue section were selected, and the score was averaged from the values acquired from each field.

### Immunohistochemistry

The Dako REAL™ EnVision™ Detection System (Dako, Glestrop, Denmark) was used for immunostaining. The tissues were first incubated with primary antibodies against IL-6, tumor necrosis factor-α (TNF-α), and IL-17 (Abcam, Cambridge, UK) overnight at 4 °C. The tissues were next incubated with a biotinylated secondary antibody, Dako REAL™ EnVision™/horseradish peroxidase was added, and the tissues were incubated for a further 30 min. The final colored product was developed using the chromogen diaminobenzidine, and the sections were examined under a photomicroscope (Olympus, Tokyo, Japan) at × 200 magnification. The number of stained cells was counted using Adobe Photoshop software (Adobe, San Jose, CA, USA) on high-power digital image (magnification × 200). Three high-powered fields per tissue section were randomly selected, and the number of stained cells was averaged from the values acquired from each field. Cells that stained positively for IL-6, TNF-α, or IL-17 were visually enumerated by four individuals (SMK, SHH, JWC, and KAJ) using higher magnification images projected onto a screen; mean values are presented.

### Confocal microscopy

Seven-micrometer-thick sections of the spleens were reacted with Alexa Fluor® 488-conjugated anti-CD4 (clone GK1.5; BioLegend, San Diego, CA, USA), phycoerythrin (PE)-conjugated anti-IL-17 (clone eBio17B7; eBioscience, San Diego, CA, USA), Alexa Fluor® 647 mouse anti-pSTAT3(pY705) (clone 4/P-STAT3; BD Pharmingen, San Diego, CA, USA), PE-conjugated anti-Foxp3 (Novus Biologicals, Littleton, CO, USA), and allophycocyanin (APC)-conjugated CD25 (clone PC61; BioLegend) antibodies. The stained sections were examined under a microscope (LSM 510 Meta; Carl Zeiss, Oberkochen, Germany) at × 200 magnification.

### Isolation of peripheral blood mononuclear cells

Mouse blood was lysed with hypotonic ACK buffer (0.15 mM NH_4_Cl, 1 mM KCO_3_, and 0.1 mM EDTA, pH 7.4). The remaining PBMCs were maintained in RPMI 1640 medium containing 5% fetal bovine serum.

### Splenocyte isolation and stimulation

Spleen cells were obtained from NOD/ShiLtJ mice and sieved through a mesh, and red blood cells were lysed in hypotonic ACK buffer. The remaining splenocytes were maintained in RPMI 1640 medium containing 5% fetal bovine serum. To induce T cell differentiation, splenocytes were stimulated with plate-bound anti-CD3 (0.5 μg/mL, BD Pharmingen) and soluble anti-CD28 antibody (1 μg/mL, eBioscience) for 72 h. For the activation of B cells, splenocytes were stimulated with lipopolysaccharide from *Escherichia coli* O111:B4 (LPS; 100 ng/mL, Sigma-Aldrich) for 72 h.

### Intracellular staining and flow cytometry

The intracellular levels of cytokines and transcription factors were assessed using anti-CD4-eFluor450 (clone RM4-5), anti-C-X-C chemokine receptor type 5 (CXCR5)–peridinin chlorophyll protein complex (PerCP)–eFluor710 (clone SPRCL5), anti-B cell lymphoma 6 (Bcl-6)–APC (clone BCL-DWN), anti-IL-17–PE (clone eBio17B7), anti-forkhead box P3 (Foxp3)–PE (clone FJK-16 s), anti-B220–APC (clone RA3-6B2), anti-CD19–PerCP–Cy5.5 (clone eBio1D3), anti-IL-10–APC (clone JES5-16E3), anti-IL-17–fluorescein isothiocyanate (FITC) (clone eBio17B7; eBioscience), anti-T and B cell activation marker (GL-7)–FITC (clone GL7; BD Pharmingen), anti-CD1d–PE (clone 1B1), and anti-CD5–FITC (clone 53-7.3; eBioscience) antibodies. In brief, the cells were stimulated for 4 h with phorbol myristate acetate (25 ng/mL) and ionomycin (250 ng/mL) in the presence of GolgiStop. Next, the cells were incubated with fixable dye (eBioscience) for 30 min and permeabilized using Cytofix/Cytoperm solution (BD Pharmingen). Thereafter, the cells were reacted with the above-listed antibodies and analyzed using a CytoFLEX flow cytometer. Events were collected and analyzed with FlowJo software (Tree Star, Ashland, CA, USA).

### Measurement of immunoglobulin G subtype levels

Blood was taken from the orbital sinuses, and serum was separated and stored at − 20 °C prior to analysis. Total immunoglobulin (Ig) G, IgG1, and IgG2a levels were measured in 100,000-fold dilutions of serum using a mouse total IgG, IgG1, and IgG2a enzyme-linked immunosorbent assay (ELISA) quantitation kit (Bethyl Lab Co., Montgomery, TX, USA). Optical densities at 450 nm were measured using an ELISA plate reader (Bio-Rad, Hercules, CA, USA).

### Real-time polymerase chain reaction

Messenger RNA (mRNA) was extracted using TRI Reagent (Molecular Research Center, Cincinnati, OH, USA) according to the manufacturer’s instructions. Complementary DNA was synthesized using the SuperScript reverse transcription system (TaKaRa, Otsu, Japan). A LightCycler 2.0 instrument (software version 4.0; Roche Diagnostics, Basel, Switzerland) was used for polymerase chain reaction amplification. All reactions were performed using LightCycler FastStart DNA Master SYBR Green I (TaKaRa) according to the manufacturer’s instructions.

The following primers were used: IL-6, 5′-AAC GAT GAT GCA CTT GCA GAA A-3′ (sense) and 5′-TCT GAA GGA CTC TGG CTT TGT C-3′ (antisense); TNF-α, 5′-ATG AGC ACA GAA AGC ATG ATC-3′ (sense) and 5′-TAC AGG CTT GTC ACT CGA ATT-3′ (antisense); IL-17, 5′-CCTCAAAGCTCAGCGTGTCC-3′ (sense), 5′-GAGCT CACTTTTGCGCCAAG-3′ (antisense); STAT3, 5′-CCG TCT GGA AAA CTG GAT AAC TTC-3′ (sense), 5′-CCT TGT AGG ACA CTT TCT GCT GC-3′ (antisense); and β-actin, 5′-GTA CGA CCA GAG GCA TAC AGG-3′ (sense) and 5′-GAT GAC GAT ATC GCT GCG CTG-3′ (antisense). All expression values were normalized by the amount of β-actin cDNA amplified from the same RNA sample and calculated by using the comparative delta-delta Ct method.

### Statistical analysis

Statistical analyses were performed using GraphPad Prism (version 5 for Windows; GraphPad Software, San Diego, CA, USA). Numerical values were compared by Student’s *t* test. Values of *p* < 0.05 were taken to indicate statistical significance.

## Results

### Metformin recovers the salivary flow rate and reduces salivary gland inflammation

**Fig. 1:**
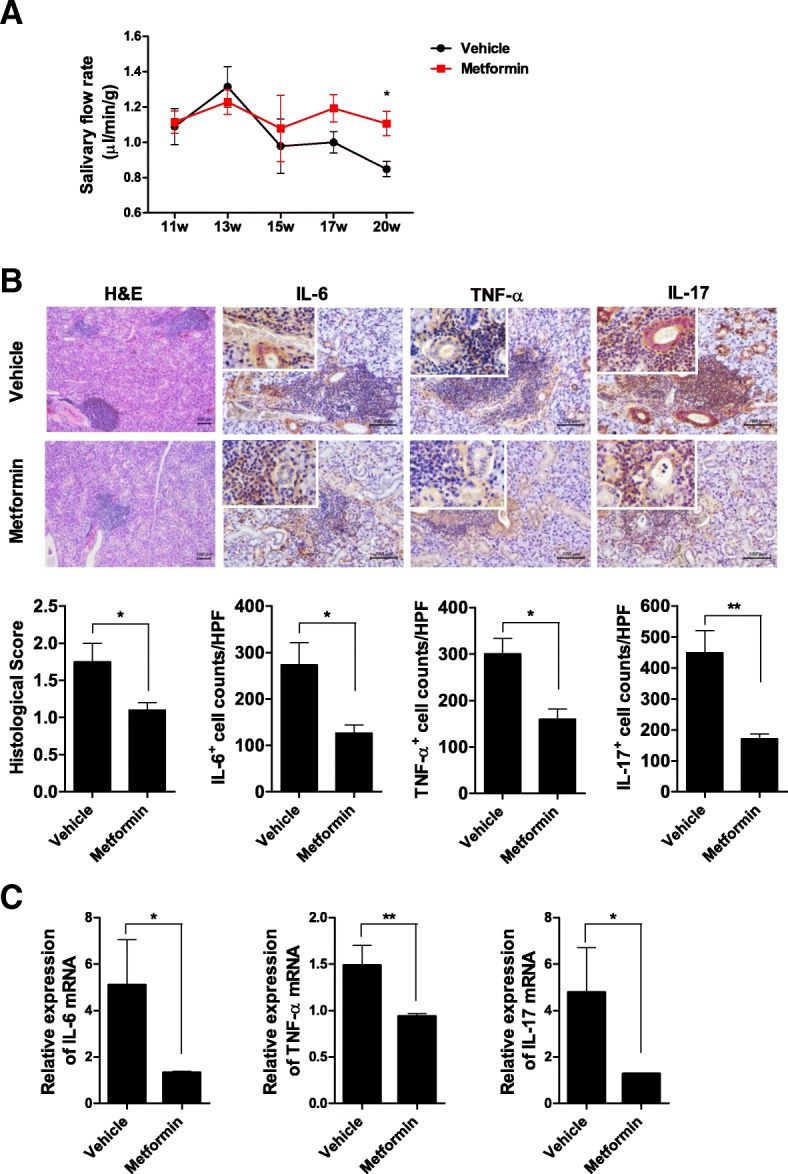
Metformin improves the salivary flow rate and salivary gland inflammation. **a** Eleven-week-old mice were orally administered vehicle or 50 mg/kg metformin daily for 9 weeks. The salivary flow rate was measured for 7 min at 11, 13, 15, 17, and 20 weeks (*n* = 5 per group at each time point). Symbols indicate means, and bars indicate SEMs. Data are representative of the two independent experiments. **b** Histological analysis of the salivary glands from vehicle- and metformin-treated mice (at 20 weeks of age, *n* = 5 per group) was conducted by hematoxylin and eosin staining (original magnification, × 100) and immunohistochemical staining for IL-6, TNF-α, and IL-17 (original magnification, × 200; insets, × 400). Histological score and numbers of IL-6-, TNF-α-, and IL-17-expressing (positive) cells are shown; scale bar, 100 μm. **c** IL-6, TNF-α, and IL-17 mRNA levels in the salivary glands, as determined by real-time PCR. Data are means ± SEMs. Data are representative of three independent experiments (*p < 0.05, **p < 0.01)

The salivary flow rate did not differ between mice treated with metformin and vehicle at baseline (week 11). The salivary flow rates decreased in vehicle-treated mice from weeks 11 to 20, but the salivary flow rates did not decrease in metformin-treated mice from weeks 11 to 20. Salivary flow rate was significantly low in vehicle-treated mice compared with metformin-treated mice at week 20 (Fig. 1a).

Histological examination of the salivary gland was performed at 20 weeks of age. Histological analysis showed that metformin reduced infiltration of lymphocytes and decreased IL-6, TNF-α, and IL-17 expression compared with the control vehicle (Fig. 1b). Moreover, metformin reduced the IL-6, TNF-α, and IL-17 mRNA levels compared with the control (Fig. 1c).

The blood glucose levels were measured in mice administered with metformin and those administered with vehicle at weeks 11, 13, and 20. Our study excluded mice which developed hyperglycemia (blood glucose ≥ 200 mg/dL) throughout the study period. Mean blood glucose levels did not differ significantly between the two groups at each time point (mean blood glucose at week 13, 114.8 and 106.2 mg/dL in vehicle- and metformin-treated mice; mean blood glucose at week 20, 115.6 and 108.4 mg/dL, respectively; see Additional file 1).

### Metformin modulates CD4^+^ T cell differentiation

**Fig. 2:**
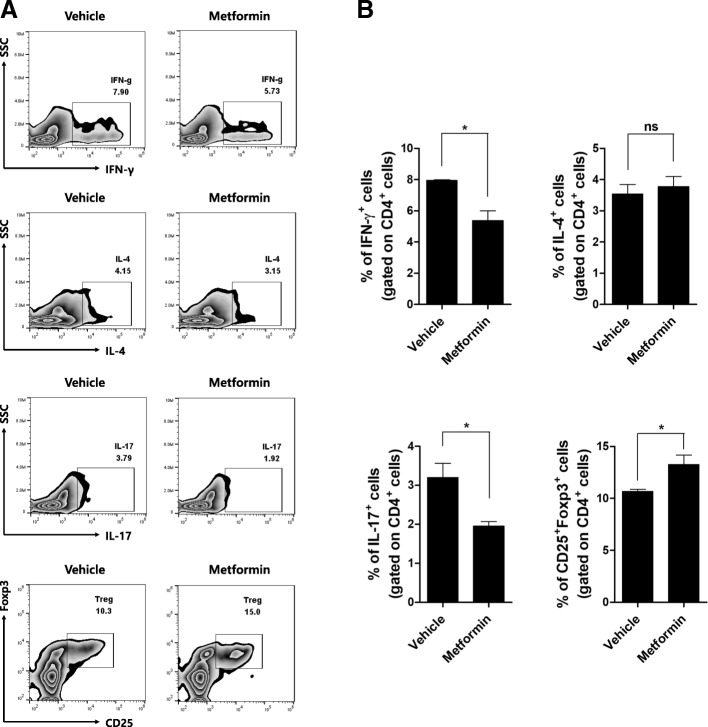
Metformin controls CD4^+^ T cell differentiation into effector or regulatory T cells in the peripheral blood. **a** Eleven-week-old mice were orally administered vehicle or 50 mg/kg metformin daily for 9 weeks (*n* = 5 per group). Next, PBMCs were stained with antibodies against CD4, IFN-γ, IL-4, IL-17, CD25, or Foxp3 and subjected to flow cytometry. Dot plots gated on CD4^+^ T cell population showed CD4^+^IFN-γ^+^ (Th1), CD4^+^IL-4^+^ (Th2), CD4^+^IL-17^+^ (Th17), and CD4^+^CD25^+^Foxp3^+^ (Treg) cells. Numbers in the plots indicate percentages of gated cells. The data were pre-gated on live single cells. **b** Data are means ± SEMs. Data are representative of three independent experiments (*p < 0.05)

Flow cytometry showed that the Th1 and Th17 cell populations in the peripheral blood were markedly decreased in metformin-treated mice compared with control mice, while the Treg cell population was significantly increased in metformin-treated mice (Fig. 2).

**Fig. 3:**
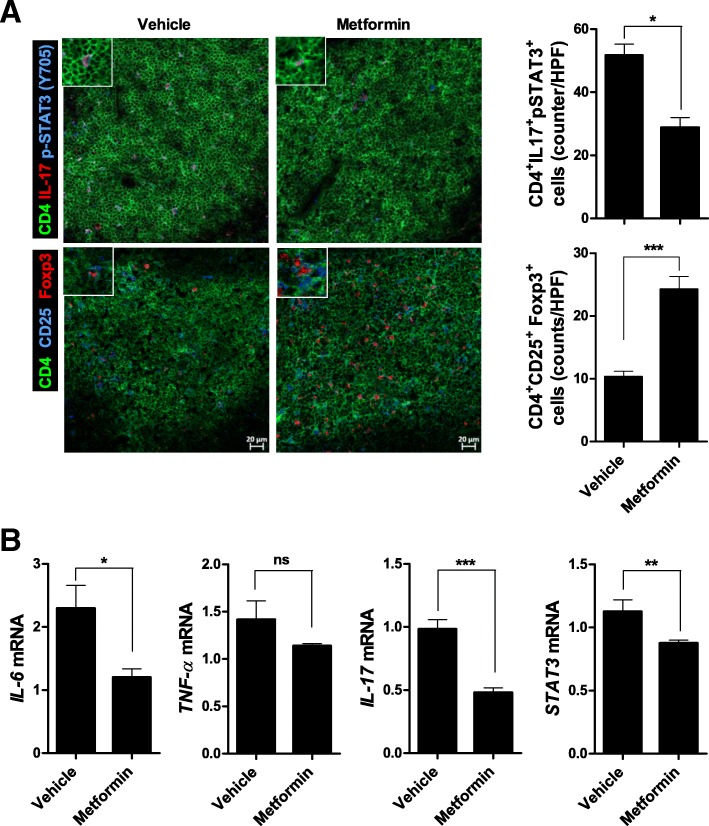
Metformin suppresses splenic Th17 cell populations and enhances Treg populations. **a** Eleven-week-old mice were orally administered vehicle or 50 mg/kg metformin daily for 9 weeks (*n* = 5 per group). The spleens were removed and reacted with antibodies against CD4, IL-17, p-STAT3 (Y705), CD25 or Foxp3. CD4^+^IL-17^+^p-STAT3 (Y705)^+^ (Th17) cells and CD4^+^CD25^+^Foxp3^+^ (Treg) cells were analyzed by confocal laser microscopy (original magnification, × 200; insets, × 400). **b** Splenic IL-6, TNF-α, IL-17, and STAT3 mRNA levels in vehicle- and metformin-administered mice, as determined by real-time PCR. Data are means ± SEMs. Data are representative of three independent experiments (*p < 0.05, **p < 0.01, ***p < 0.001)

Confocal microscopy showed that metformin reduced the splenic CD4^+^IL-17^+^pSTAT3(Y705)^+^ cell population and increased the CD4^+^Foxp3^+^CD25^+^ cell population (Fig. 3a). Moreover, metformin reduced the IL-6, IL-17, and STAT3 mRNA levels compared with the control vehicle (Fig. 3b).

### Metformin maintains the balance between Tfh and Tfr cells

**Fig. 4:**
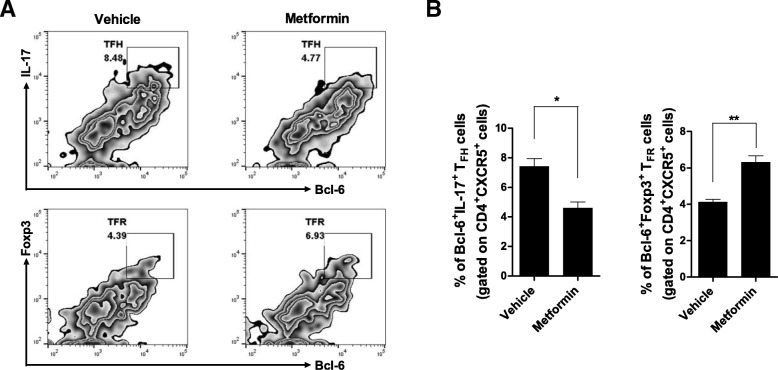
Metformin regulates the balance between Tfh cells and Tfr cells in vitro. **a** Splenocytes from NOD/ShiLtJ mice were cultured for 3 days with anti-CD3 and anti-CD28 antibodies in the presence or absence of 1 mM metformin (*n* = 3 per group). The cells were next reacted with antibodies against CD4, CXCR5, Bcl-6, IL-17, or Foxp3. The frequencies of Tfh cells (Bcl6^+^IL-17^+^ cells gated on CD4^+^CXCR5^+^ T cell population) and Tfr cells (Bcl6^+^Foxp3^+^ cells gated on CD4^+^CXCR5^+^ T cell population) are shown. Numbers in the plots indicate percentages of gated cells. The data were pre-gated on live single cells. **b** Data are means ± SEMs. Data are representative of three independent experiments (*p < 0.05, **p < 0.01)

Spleen cells were isolated from the NOD/ShiLtJ mice and cultured in vitro for 72 h with anti-CD3 and anti-CD28 antibodies with or without 1 mM metformin. Metformin decreased T cell differentiation into CD4^+^CXCR5^+^Bcl-6^+^IL-17^+^ cells and increased T cell differentiation into CD4^+^CXCR5^+^Bcl-6^+^Foxp3^+^ cells (Fig. 4).

### Metformin controls B cells differentiation

**Fig. 5:**
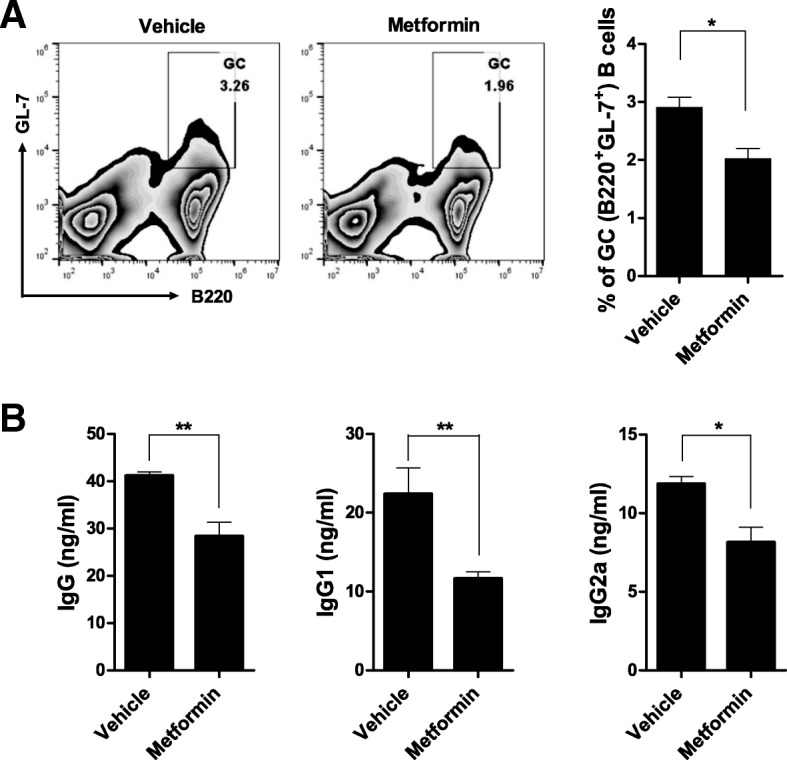
Metformin suppresses Ig isotype switching and B cell differentiation into GC B cells. **a** Eleven-week-old mice were orally administered vehicle or 50 mg/kg metformin daily for 9 weeks (*n* = 5 per group). PBMCs were isolated, reacted with antibodies to B220 and GL-7, and subjected to flow cytometry to identify B220^+^GL7^+^ GC B cells. The data were pre-gated on live single cells. **b** Serum total IgG, IgG1, and IgG2a levels in NOD/ShiLtJ mice administered with metformin (*n* = 5) or vehicle (*n* = 5), as determined by ELISA (mouse serum was diluted 100,000-fold). Data are means ± SEMs. Data are representative of three independent experiments (*p < 0.05, **p < 0.01)

Metformin suppressed B cell differentiation into GC B (B220^+^GL-7^+^) cells compared with the control vehicle (Fig. 5a). It reduced serum total IgG and IgG subtype (IgG1 and IgG2a) levels, suggesting decreased Ig isotype switching (Fig. 5b).

**Fig. 6:**
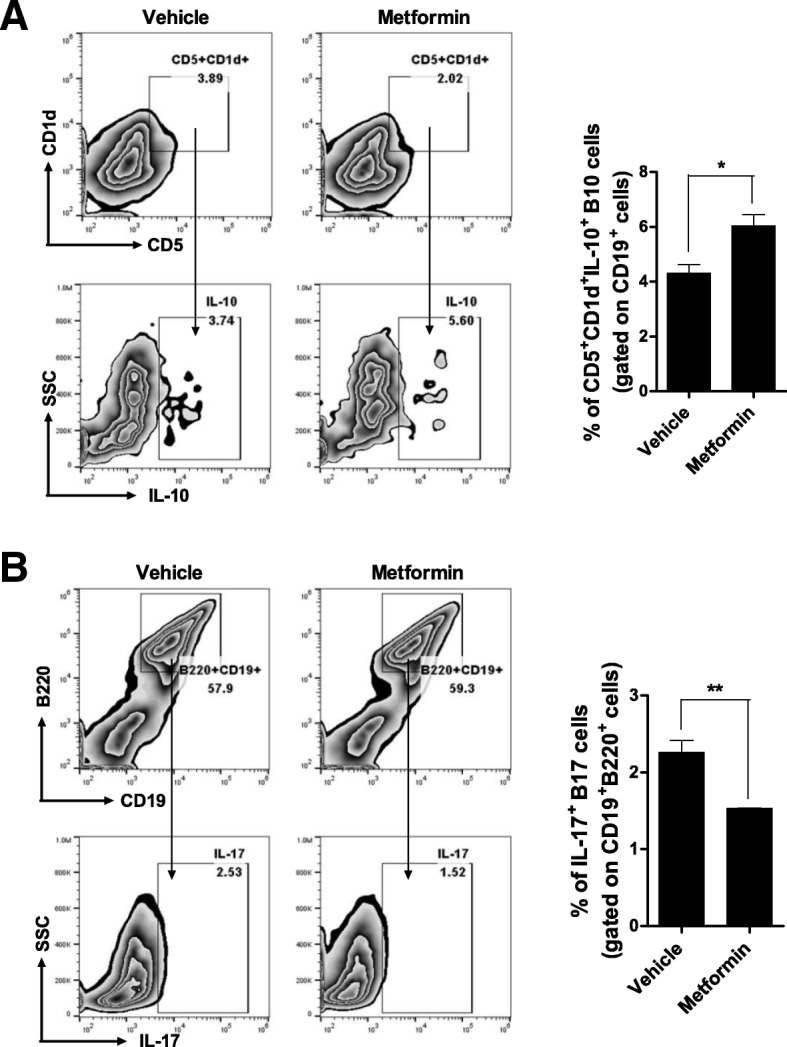
Metformin regulates the balance between IL-10- and IL-17-producing B cells in vitro. **a**, **b** Splenocytes from NOD/ShiLtJ mice were cultured for 3 days with LPS in the presence or absence of 1 mM metformin (*n* = 3 per group) and subsequently reacted with antibodies against B220, CD19, CD5, CD1d, IL-10, and IL-17. The frequencies of B10 cells (CD5^+^ CD1d^+^IL-10^+^ cells gated on CD19^+^ cell population) and B17 cells (IL-17^+^ cells gated on CD19^+^B220^+^ cell population) were analyzed. The data were pre-gated on live single cells. Data are means ± SEMs. Data are representative of three independent experiments (*p < 0.05, **p < 0.01)

Spleen cells from NOD/ShiLtJ mice were cultured in vitro for 72 h with LPS with or without metformin. B cell differentiation into IL-10- or IL-17-producing B cells was assessed by flow cytometry. Metformin-treated splenocytes were frequently differentiated to IL-10-producing (CD19^+^CD5^+^CD1d^+^IL-10^+^) than IL-17-producing (CD19^+^B220^+^ IL-17^+^) B cells compared with vehicle-treated splenocytes (Fig. 6).

## Discussion

In this study, metformin improved salivary gland inflammation and the salivary flow rate without altering the serum glucose level in a mouse model of SS. Metformin suppressed CD4^+^ T cell differentiation into Th17, Th1, and Tfh cells and enhanced that into Treg and Tfr cells in vivo and in vitro. Metformin also controlled B cell differentiation by reducing GC B cell population and serum IgG levels, in addition to promoting the balance between IL-10- and IL-17-producing B cells. These effects of metformin are mediated by the inhibition of mTOR-STAT3 via AMPK activation [9, 10, 32, 33].

The mTOR pathway is crucial for determining antigen-stimulated CD4^+^ T cell differentiation into effector or regulatory lineage cells [14, 34–36]. Under transforming growth factor-β and IL-6 stimulation, CD4^+^ T cells differentiate into Th17 cells, which express STAT3 and RAR-related orphan receptor gamma. mTOR is involved in the integration of the cytokine signals and activation of the transcription factors required for the development of specific T cell subsets [14]. mTOR-deficient T cells, in which the appropriate transcription factors are not activated, preferentially differentiate into Treg cells rather than Th1, Th2, or Th17 cells [14]. Metformin targets mainly mTORC1, which is highly activated in Th1 and Th17 cells [34, 37]. This targeting may explain metformin’s downregulation of Th1 and Th17 cells and upregulation of Treg cells in this study. CD4^+^ T cells, particularly Th1 and Th17 cells, infiltrate the exocrine glands of patients with SS and modulate disease severity [38, 39]. Therefore, regulation of CD4^+^ T cell differentiation by metformin may modulate the pathogenesis and outcomes of SS.

Indeed, available mTOR inhibitors showed therapeutic potential for autoimmune diseases including SS. Although the results were derived from a study using an animal model of SS, rapamycin, an mTOR inhibitor, was effective to suppress autoimmune dacryoadenitis [27]. Moreover, a study published as an abstract showed that rapamycin decreased B cell proliferation and IgG production in patients with SS [40].

GC-like structures, as well as glandular inflammation, are observed in the exocrine glands of patients with SS. GC-like structures have autoimmune features such as T and B cell segregation, follicular dendritic cells, chemokines, and adhesion molecules [41]. These GC-like structures in target organs are related to the production of autoantibodies (anti-Ro/SSA and anti-La/SSB), loss of gland function, and an elevated risk of lymphoma [41, 42]. Importantly, Tfh cells and B cells play essential roles in these structures by driving functional GC responses [43]. Indeed, Tfh cell population is increased in patients with SS, particularly in those with severe disease [44, 45]. STAT3, which is downstream of mTORC1, is involved in the development of Tfh cells. STAT3-deficient CD4^+^ T cells are defective in Tfh cell differentiation, leading to decreased GC B cell development [18]. Additionally, IL-21/IL-21R/STAT3 signaling is required for the differentiation of naïve B cells into antibody-producing plasma cells [19, 20]. These findings suggest that metformin suppresses Tfh and B cell differentiation and controls the abnormal GC responses in SS by inhibiting STAT3.

STAT3 mediates cytokine signaling and promotes the immune response. In patients with SS, STAT3 is activated and inflammatory cytokines are overexpressed in salivary gland tissues and PBMCs. The Th17-associated cytokines IL-23 and IL-17 are highly expressed in the salivary glands of patients with SS via IL-6-induced STAT3 pathway [25]. IL-6/STAT3 signaling also reportedly induces the expression of a new autoantigen in salivary gland epithelial cells from patients with SS [46]. However, neither Janus kinase 1 nor tyrosine kinase 2 (upstream of STAT3) is activated, whereas STAT3 is constitutively activated, in SS [23]. Thus, STAT3 activation in SS may be due to the impaired STAT3 suppressor function, rather than the augmented cytokine signaling. Indeed, suppressor of cytokine signaling 3, a negative regulator of STAT3, is reportedly functionally impaired in SS, leading to STAT3/IL-17 pathway activation [26]. A recent study revealed that Act1 was a negative regulator of STAT3, and Act1-deficient mice represented SS-like disease by affecting IL-23- and IL-21-induced STAT3 activation in T and B cells [47]. Therefore, STAT3-targeted agents might be effective against SS.

Metformin exerted anti-inflammatory and immunomodulatory effects with no significant glucose-lowering effect in an animal model of SS. Determining the optimum metformin dose for humans might be challenging. However, we found that a relatively low dose of metformin (human equivalent dose of 4 mg/kg/day) could be effective for SS without developing hypoglycemia. AMPK was activated by long-term, but not short-term, metformin treatment [5]. Because the anti-inflammatory and immunomodulatory effects of metformin are represented via activation of AMPK, development of drugs for SS should take into consideration the potential activation of AMPK under optimal dose and long-term metformin treatment.

A limitation of our study is that we conducted the study using an animal model of secondary SS. Female NOD mice developed diabetes with an incidence of 60% by 16 weeks of age and incidence of 74% by 26 weeks of age [48, 49]. The present study excluded the mice which developed hyperglycemia during the study period to show the therapeutic effect of metformin against SS. However, metformin might have affected the incidence of diabetes during the study period, being a confounding to the therapeutic effect of metformin. Further research using a primary SS model, such as C57BL/6.NOD-*Aec1Aec2*, is required [50].

## Conclusions

Metformin reduced salivary gland inflammation and restored the salivary flow rate in an animal model of SS. Metformin modulates clinical and histologic aspects of SS by maintaining the balance between effector T and regulatory T cells and by controlling B cell differentiation. Therefore, metformin may be a potential therapeutic for SS.

## Additional file

Additional file 1: Comparison of blood glucose levels between mice administered with metformin and vehicle. The blood glucose levels were measured in mice administered with metformin and those administered with vehicle at weeks 11, 13, and 20 (*n* = 5 per group at each time point). Mean blood glucose levels did not differ significantly between the two groups at each time point (mean blood glucose at week 13, 114.8 and 106.2 mg/dL in vehicle- and metformin-treated mice; mean blood glucose at week 20, 115.6 and 108.4 mg/dL, respectively). Data are presented as the mean ± SEM. (JPG 148 kb)
