# Supplementary figures and images for: Metformin improves salivary gland inflammation and hypofunction in murine Sjögren’s syndrome
Source: Arthritis Res Ther. 2019 Jun 4;21:136. doi: 10.1186/s13075-019-1904-0 (PMC6549273; doi:10.1186/s13075-019-1904-0)

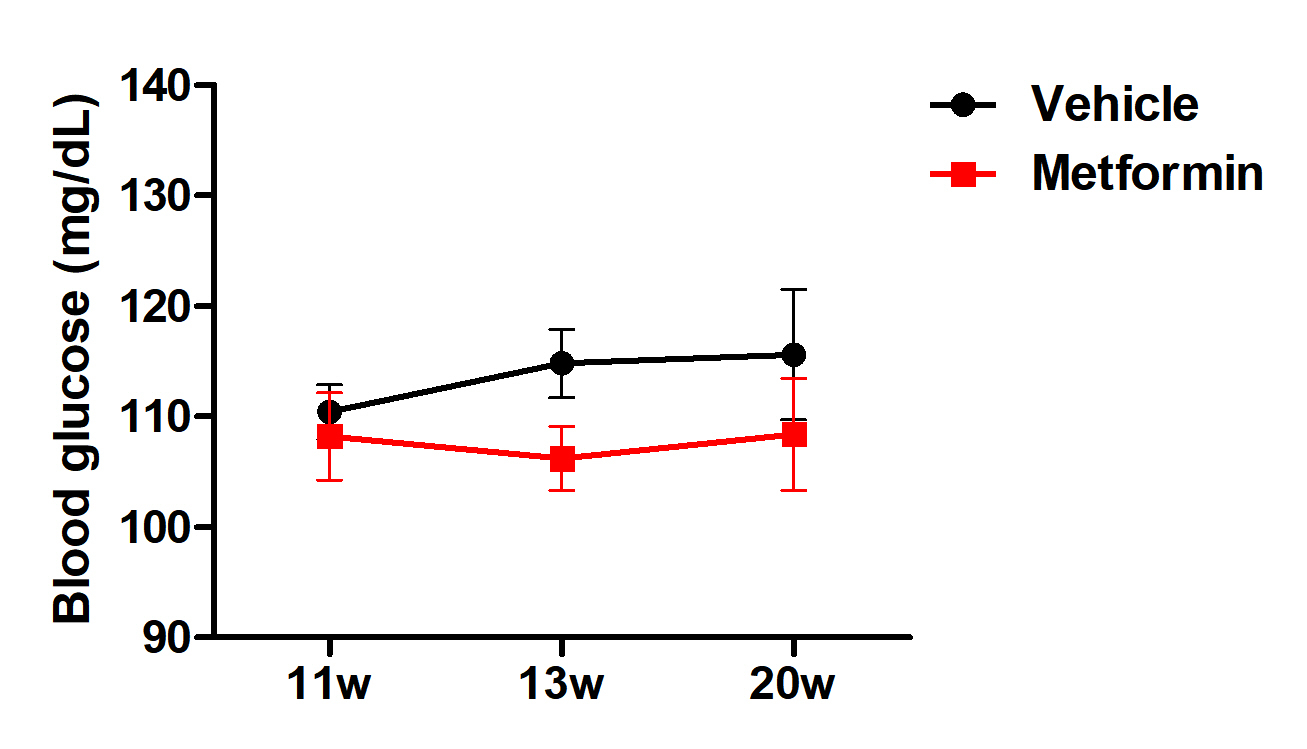

Supplement: Supplementary file 1 — Comparison of blood glucose levels between mice administered with metformin and vehicle. The blood glucose levels were measured in mice administered with metformin and those administered with vehicle at weeks 11, 13, and 20 (n = 5 per group at each time point). Mean blood glucose levels did not differ significantly between the two groups at each time point (mean blood glucose at week 13, 114.8 and 106.2 mg/dL in vehicle- and metformin-treated mice; mean blood glucose at week 20, 115.6 and 108.4 mg/dL, respectively). Data are presented as the mean ± SEM. (JPG 148 kb) [file 13075_2019_1904_MOESM1_ESM.jpg]
